# Supplementary material for: A Rapid Approach for Identifying Cell Lines Lacking Functional Cytidine Deaminase
Source: Int J Mol Sci. 2025 Apr 3;26(7):3344. doi: 10.3390/ijms26073344 (PMC11989883; doi:10.3390/ijms26073344)
Supplement: Supplementary file 1 [file ijms-26-03344-s001.zip › ijms-3510260-supplementary.pdf]

Supplementary Materials

A Rapid Approach for Identifying Cell Lines Lacking Functional Cytidine Deaminase

Anna Ligasová [1,*], Markéta Kociánová [2] and Karel Koberna [1,*]

[1] Institute of Molecular and Translational Medicine, Faculty of Medicine and Dentistry and Czech Advanced Technology and Research Institute, Palacký University Olomouc, Hněvotínská 5, 779 00 Olomouc, Czech Republic

[2] Institute of Molecular and Translational Medicine, Faculty of Medicine and Dentistry, Palacký University Olomouc, Hněvotínská 5, 779 00 Olomouc, Czech Republic

* Correspondence: anna.ligasova@upol.cz (AL)), karel.koberna@upol.cz (KK)

Supplementary Figure S1

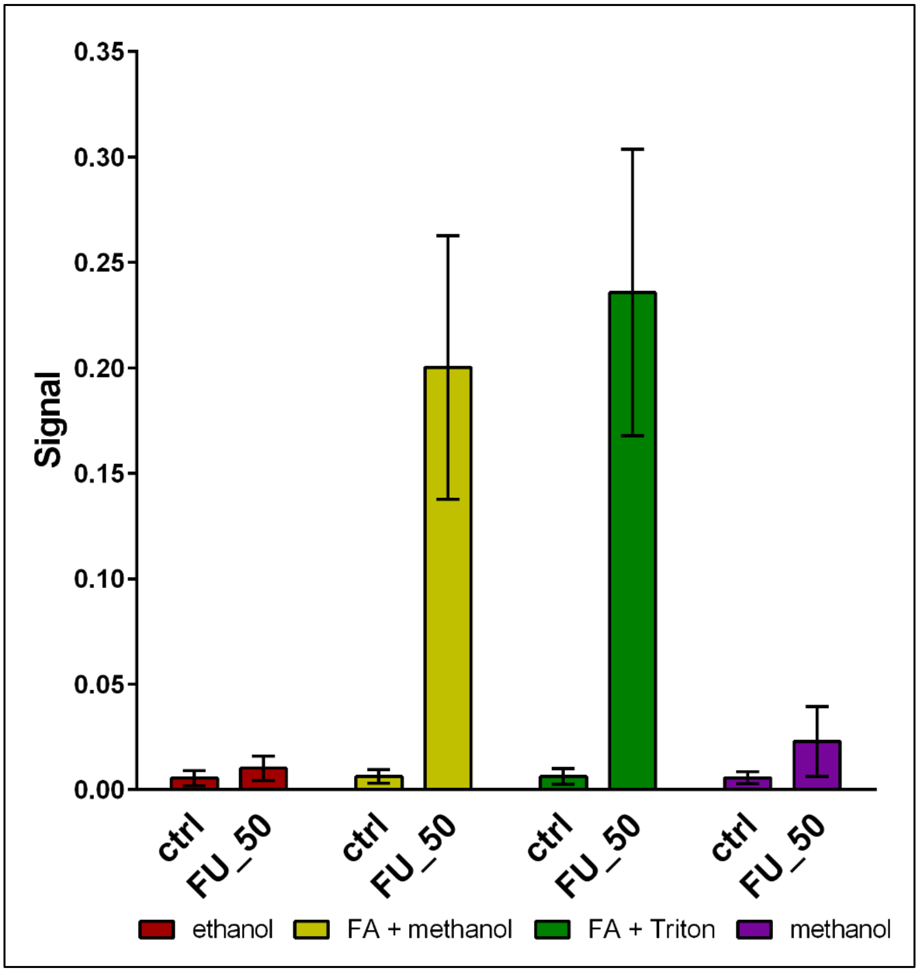

Supplementary Figure S1. Comparison of various fixation and permeabilization protocols for FU detection
HeLa cells were incubated with or without 50 µM FU, then fixed and permeabilized using four different protocols. Incorporated FU was subsequently detected. Data are presented as mean ± SD.
